# Prophylactic pirfenidone to prevent radiation- induced lung injury in ICI-pretreated lung cancer patients: a phase II study

**DOI:** 10.3389/fonc.2026.1876642

**Published:** 2026-10-15

**Authors:** Yujie Huang, Jia Ma, Guang Han, JianPing Bi, Guoliang Pi, Ying Li, Yi Peng, Chuangying Xiao, Xiulin Tuo

**Affiliations:** 1 Department of Radiation Oncology, Hubei Cancer Hospital, Tongji Medical College, Huazhong University of Science and Technology, Wuhan, China; 2 Department of Oncology, Jianghan Oilfield General Hospital, Qianjiang, China

**Keywords:** immune checkpoint inhibitors, lung cancer, pirfenidone, radiation-induced lung injury, thoracic radiotherapy

## Abstract

**Objective:**

This study aimed to relieve radiation-induced lung injury (RILI) by assessing the use of prophylactic pirfenidone. Subjects were lung cancer patients with prior ICI treatment who received thoracic radiotherapy.

**Methods:**

Patients with lung cancer were enrolled in this phase II single-arm study. All candidates had a history of prior ICI exposure (≥2 cycles; interval ≤6 months) scheduled for thoracic radiotherapy (≥50 Gy). Patients received oral pirfenidone (1800 mg/day) from one week pre-radiotherapy to four weeks post-completion. The primary endpoint was the 6-month incidence of ≥grade 2 RILI, compared to a 40% historical control (p_0_=0.40; p_1_=0.22; α=0.10; power=80%). Secondary endpoints included pulmonary function (DLCO%, FVC%) and safety.

**Results:**

A total of 39 patients were enrolled in this trial. At the 6-month follow-up, the incidence of grade 2 or higher RILI was 23.1% (9/39; 95% CI: 11.1%–39.3%). This rate was statistically significantly lower than the 40% incidence from the historical control cohort (p=0.0205). Grade 3 RILI occurred in 7.7% (3/39); no grade 4–5 events occurred. Mean DLCO% declined by 7.6 ± 5.4% at 3 months and 6.5 ± 5.8% at 6 months. The pirfenidone completion rate was 84.6% (median relative dose intensity: 89.3%). Common adverse events were fatigue (43.6%), nausea (41.0%), and appetite loss (33.3%), mostly grade 1–2.

**Conclusions:**

Preventive use of pirfenidone was associated with a lower observed incidence and severity of RILI than the predefined historical control in ICI-pretreated lung cancer patients receiving thoracic radiotherapy. Additionally, the intervention showed a potential protective trend for pulmonary diffusion function. These findings require further validation in a phase III trial.

## 1 Introduction

The integration of immune checkpoint inhibitors (ICIs) into the multimodality management of lung cancer, particularly as consolidation therapy following chemoradiotherapy, has significantly improved survival outcomes (1). Consequently, an increasing proportion of patients are receiving thoracic radiotherapy after prior ICI exposure (2, 3). However, this sequential therapeutic paradigm presents a formidable clinical challenge: the compounded risk of severe pulmonary toxicity. Prior ICI therapy can prime the pulmonary microenvironment, leading to an exaggerated inflammatory and fibrotic response upon subsequent radiation exposure (4, 5).

Latest clinical data highlight the seriousness of this risk. Symptomatic radiation-induced lung injury (RILI) is a major health risk for lung cancer patients who have used immune checkpoint inhibitors (ICI) before. The incidence rate is over 45 percent. This risk is even higher, particularly among those with a high dose of lung radiation or a history of immune-related adverse events (6–8). This high-risk group has a great chance to develop RILI. But there are still no approved drugs for prevention in clinical practice. Current clinical care mainly uses a passive treatment model. Management remains largely reactive, relying on high-dose systemic corticosteroids after symptom onset (9, 10).

The development of RILI is caused by a series of inflammatory and fibrotic processes. These biological processes are mainly controlled by the transforming growth factor-beta (TGF-β) signaling pathway (11–13). Pirfenidone is an orally administered antifibrotic agent with high bioavailability, which has been officially approved for idiopathic pulmonary fibrosis. Its core mechanism lies in suppressing the production of TGF-β (14, 15). In preclinical models, pirfenidone may attenuate radiation-induced pulmonary fibrosis (16, 17). More importantly, accumulating clinical results have proved the good clinical value of this drug. Pirfenidone can improve lung function in patients with grade 2 to 3 RILI. It can also help lung lesions fade away (18).

Based on the above mechanistic basis and preliminary clinical findings (19, 20), we hypothesized that starting prophylactic administration of pirfenidone ahead of thoracic radiotherapy may block subsequent fibrotic cascades. This strategy may further decrease RILI occurrence in patients with prior ICI exposure. We designed and carried out a phase II single-arm trial. The study aimed to assess the efficacy and safety of prophylactic administration of pirfenidone in this high-risk population, aiming to provide proof-of-concept data for future confirmatory studies.

## 2 Methods

### 2.1 Study design

This clinical trial was designed as a single-center, single-arm and open-label phase II study. Simon’s optimal two-stage statistical design was adopted for trial arrangement (21). The Institutional Ethics Committee granted approval for this study (Approval No. LCKY2023011). All research procedures strictly followed the principles of the Declaration of Helsinki and ICH-GCP guidelines. Every participant signed written informed consent prior to study enrollment. The trial was prospectively registered at ClinicalTrials.gov (NCT05801133).

### 2.2 Participants

Study participants were adult patients aged 18 to 75 years. All had histologically or cytologically confirmed primary lung cancer, including NSCLC and SCLC subtypes and received definitive or palliative thoracic radiotherapy. Key inclusion criteria comprised: (1) prior treatment with at least two cycles of an anti-PD-1 or anti-PD-L1 antibody; (2) an interval of ≤6 months between the last ICI dose and the initiation of radiotherapy; (3) a planned total radiation dose of ≥50 Gy; (4) ECOG performance status was maintained at grade 0 or 1 for all enrolled subjects; and (5) adequate baseline pulmonary function (FEV1 > 1.0 L, DLCO > 40% predicted).

Key exclusion criteria: (1) a history of ≥grade 2 ICI-related pneumonitis or prior symptomatic radiation pneumonitis; (2) severe baseline interstitial lung disease or active pulmonary infection; (3) Patients with serious hepatic damage (ALT/AST > 3-fold upper normal limit) or impaired renal function (eGFR < 30 mL/min per 1.73m²); (4) known hypersensitivity to pirfenidone; and (5) concurrent systemic corticosteroids exceeding 10 mg/day prednisone equivalent at baseline.

The ≤6-month interval between the last ICI dose and radiotherapy initiation was prespecified as a pragmatic operational definition rather than an established pharmacodynamic threshold, in order to enrich for patients whose pulmonary microenvironment might still be immunologically primed at the time of irradiation, a group in which symptomatic pneumonitis rates after thoracic radiotherapy are consistently elevated (6–8). To characterize heterogeneity in this variable, the ICI-to-radiotherapy interval and the number of prior ICI cycles were recorded for every patient and the interval was examined as a prespecified exploratory subgroup (≤60 vs. >60 days).

### 2.3 Treatment protocol

Oral pirfenidone was administered following a two-week dose titration schedule (600 mg/day in week 1, 1200 mg/day in week 2) and escalated to the target dose of 1800 mg/day (600 mg three times daily with meals) from week 3 onward. Treatment was initiated one week before the start of thoracic radiotherapy and continued throughout the radiotherapy course and for four weeks after its completion (total planned duration: approximately 11 weeks). Dose reductions (to 1200–1600 mg/day) or temporary interruptions were permitted for unacceptable toxicity, including grade ≥2 gastrointestinal toxicity, photosensitivity, or hepatotoxicity. Thoracic irradiation was delivered using intensity-modulated radiotherapy (IMRT) or volumetric modulated arc therapy (VMAT). The prescription dose was ≥50 Gy in conventional fractionation (1.8–2.0 Gy/fraction). Dose-volume constraints were set for lung tissue. The constraints included V20 ≤ 30%, V5 ≤ 60%, and mean lung dose (MLD) ≤ 15 Gy. Concurrent chemotherapy was allowed based on the clinical judgment of attending physicians. Detailed information on specific treatment regimens is presented in the Results section.

To minimize anticipated toxicity, pirfenidone was up-titrated over two weeks and taken with food to improve gastrointestinal tolerability, and all patients received proactive supportive care, including counselling on photoprotection (sun avoidance, protective clothing, and broad-spectrum sunscreen) together with antiemetic and dermatological measures as required. Concurrent immune checkpoint inhibitor therapy was not permitted during radiotherapy or during the pirfenidone treatment period; no patient received ICI during these phases. Decisions regarding post-radiotherapy systemic therapy, including resumption of or consolidation with ICIs, were made at the discretion of the treating physician after completion of the radiotherapy course and were recorded.

### 2.4 Endpoints and assessments

The primary endpoint was the cumulative rate of grade 2 or higher radiation-induced lung injury (RILI) within six months after radiotherapy. All adverse event grading followed the Common Terminology Criteria for Adverse Events (CTCAE 5.0). To mitigate assessment bias inherent to the open-label design, RILI diagnosis and grading were adjudicated by an independent review panel comprising at least two radiation oncologists and one pulmonologist. All panel experts independently assessed patients’ clinical symptoms and high-resolution computed tomography (HRCT) imaging features and evaluated the clinical requirement for corticosteroid therapy (22); discordant assessments were resolved by consensus discussion. Key secondary endpoints included: (1) alterations in predicted DLCO (% predicted) and FVC (% predicted) at 3 and 6 months after radiotherapy completion; (2) incidence of grade ≥3 RILI; (3) median time to onset of grade ≥2 RILI; and (4) safety and tolerability of pirfenidone, assessed according to CTCAE v5.0. Exploratory endpoints comprised subgroup analyses of the primary endpoint stratified by lung V20 (≥20% vs. <20%), ICI-to-radiotherapy interval (≤60 days vs. >60 days), concurrent chemotherapy status, and prior grade 1 checkpoint inhibitor pneumonitis (CIP); disease stage was examined descriptively.

Radiation-induced lung injury (RILI) is used as the overarching term encompassing the acute–subacute inflammatory phase (radiation pneumonitis) and subsequent radiation fibrosis; checkpoint inhibitor pneumonitis (CIP), also termed immune-related pneumonitis, denotes pneumonitis attributable to prior ICI therapy; and radiation recall pneumonitis denotes inflammation recurring within a previously irradiated volume after a subsequent systemic-therapy trigger.

RILI was diagnosed when new respiratory symptoms and/or radiographic changes developed after radiotherapy and were judged attributable to radiation rather than to an alternative aetiology. Supportive features included ground-glass or consolidative opacities with a spatial distribution conforming to the radiation dose distribution (typically non-anatomical and corresponding to the high-dose region), onset within the expected temporal window (generally 1–6 months after radiotherapy), and, for symptomatic events, new or worsening dyspnea, cough, or low-grade fever meeting CTCAE v5.0 grade ≥2 criteria (grade 2 additionally requiring corticosteroids). Before RILI was assigned, alternative causes were actively excluded, comprising infection (clinical assessment, inflammatory markers, and microbiological testing where indicated), tumor progression (comparison with the disease distribution on serial imaging), cardiogenic pulmonary edema, and chemotherapy-related lung injury. Events with a diffuse, bilateral, or non-dosimetric distribution occurring in the appropriate temporal context were classified as CIP rather than RILI, and radiation recall pneumonitis was recorded separately. Each event was scored independently by every panel member; any discordance in diagnosis or grade was resolved by joint review of the imaging and consensus discussion, with the CTCAE v5.0 definitions applied as the final arbiter.

### 2.5 Statistical design and analysis

#### 2.5.1 Justification of the historical control rate (p_0_)

The null hypothesis rate of 40% was based on published data from two contemporary studies of ICI-pretreated or ICI-era lung cancer populations. *Xiao* et al. (2025) reported a 45.07% (32/71) incidence of symptomatic radiation pneumonitis in the ICI-pretreated subgroup of a multicenter prospective biomarker cohort study of 214 lung cancer patients receiving thoracic radiotherapy (6). *Yegya-Raman* et al. (2024) documented a 1-year cumulative incidence of ≥ grade 2 pneumonitis of 31.4% in 335 patients treated in the immunotherapy consolidation era (7). Based on these data, p_0_ = 0.40 was selected as a conservative estimate positioned between the two reported rates. A detailed comparison of baseline characteristics between these historical cohorts and the present study is provided in **Supplementary Table 1**.

#### 2.5.2 Simon’s optimal two-stage design

The alternative hypothesis assumed that pirfenidone can lower the incidence of grade 2 or higher RILI to 22 percent (p_1_=0.22), representing an absolute risk reduction of 18 percentage points. This study set the one-sided type I error rate (α) at 0.10 in advance. Phase III confirmatory clinical trials usually use a significance level of 0.05. By contrast, phase II proof-of-concept studies with a Simon design widely accept the level of α=0.10. This setting can minimize the risk of wrongly ruling out potentially effective treatments when the sample size is limited (21). The statistical power (1−β) was set at 80%. Under this premise, Simon’s optimal two-stage design yielded the following study parameters. Stage 1 required n_1_= 19 evaluable patients. If ≥ 7 RILI events were observed (i.e., exceeding the futility boundary of a_1_= 6), the trial would be terminated for futility. By contrast, if the number of RILI events was 6 or fewer, another 20 patients (resulting in 39 participants in total) could be enrolled and would proceed to Stage 2. When the total RILI event count in all 39 study participants was 11 or fewer, the null hypothesis would be rejected, corresponding to an observed incidence rate below 28.2%.

#### 2.5.3 Statistical methods

The primary endpoint was analyzed using the exact Clopper-Pearson method to calculate 95% confidence intervals and a one-sided exact binomial test against p_0_ = 0.40. We assessed changes in pulmonary function parameters from baseline using paired t-tests, supplemented by Wilcoxon signed-rank tests for non-normal data. Subgroup differences were compared using Fisher’s exact test. All statistical analyses were run and all figures were generated in Python 3.11 (SciPy, v1.11; Matplotlib, v3.8). For the primary endpoint, a one-sided α of 0.10 was used as predefined by the Simon’s two-stage design. For secondary endpoints, a two-sided p < 0.05 was considered statistically significant.

## 3 Results

### 3.1 Patient disposition and baseline characteristics

From June 2022 to October 2023, 52 patients were screened for eligibility, of whom 39 were enrolled (**Figure 1**). Following the Simon two-stage design, 19 patients were enrolled in the first stage. Interim analysis identified four events of ≥grade 2 RILI (21.1%). This figure did not cross the predefined futility boundary (a_1_ = 6). The trial thus advanced to the second stage, with another 20 patients enrolled.

**Figure 1:**
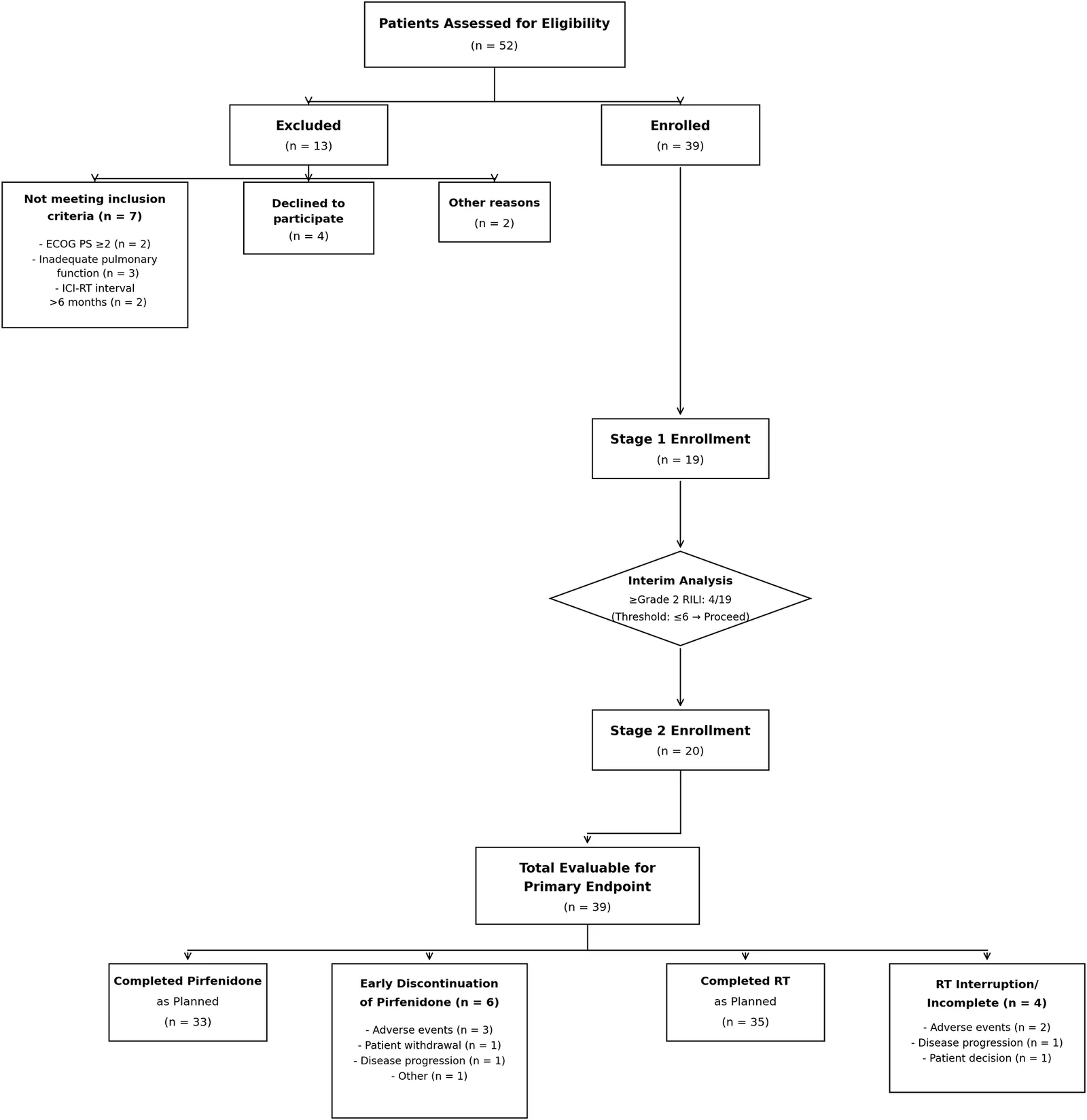
Patient disposition flowchart per the CONSORT guidelines. Boxes show the numbers of patients screened, excluded (with reasons), enrolled in each stage of Simon’s optimal two-stage design, and evaluable for the primary endpoint, together with the delivery of pirfenidone and radiotherapy. The diamond denotes the prespecified interim futility analysis performed after stage 1. ECOG PS, Eastern Cooperative Oncology Group performance status; ICI, immune checkpoint inhibitor; RILI, radiation-induced lung injury; RT, radiotherapy.

Patient baseline characteristics are outlined in **Table 1**. The enrolled cohort had a mean age of 60.1 years, with a standard deviation of 7.8, and 59.0% were male. Most participants scored 1 on the ECOG performance scale (51.3%). Meanwhile, 43.6% of patients were pathologically diagnosed with adenocarcinoma. Notably, patients had received a median of 5 cycles (range 2–10) of prior ICI therapy. The median time gap between the final ICI administration and radiotherapy initiation was 77 days, with a span of 14 to 142 days. The mean lung V20 was 21.0% (SD 4.3%) and the mean lung dose (MLD) was 8.8 Gy (SD 1.4). Concurrent chemotherapy was administered to 71.8% (28/39) of the cohort.

**Table 1:** Baseline demographics and clinical characteristics (N = 39).

Characteristic	Value
Demographics	
Age, years, mean ± SD	60.1 ± 7.8
Male sex, n (%)	23 (59.0)
Smoking status, n (%)	
Current smoker	10 (25.6)
Former smoker	15 (38.5)
Never smoker	14 (35.9)
ECOG PS, n (%)	
0	19 (48.7)
1	20 (51.3)
Tumor characteristics	
Histology, n (%)	
Adenocarcinoma	17 (43.6)
Squamous cell carcinoma	12 (30.8)
Other NSCLC	2 (5.1)
SCLC	8 (20.5)
Clinical stage, n (%)	
IIIA	11 (28.2)
IIIB	11 (28.2)
IIIC	10 (25.6)
IV	7 (17.9)
Prior ICI treatment	
ICI type, n (%)	
Pembrolizumab	10 (25.6)
Camrelizumab	10 (25.6)
Tislelizumab	8 (20.5)
Sintilimab	4 (10.3)
Nivolumab	5 (12.8)
Atezolizumab	2 (5.1)
Prior ICI cycles, median (range)	5 (2–10)
ICI–RT interval, days, median (range)	77 (14–142)
Prior grade 1 CIP history, n (%)	5 (12.8)
Radiotherapy parameters	
Prescription dose, Gy, n (%)	
50–56 Gy	10 (25.6)
60 Gy	16 (41.0)
63–66 Gy	13 (33.3)
RT technique, n (%)	
IMRT	15 (38.5)
VMAT	24 (61.5)
Concurrent chemotherapy, n (%)	28 (71.8)
V_20_, %, mean ± SD	21.0 ± 4.3
V_5_, %, mean ± SD	46.3 ± 7.0
MLD, Gy, mean ± SD	8.8 ± 1.4
Baseline pulmonary function	
DLCO, % predicted, mean ± SD	82.6 ± 13.7
FVC, % predicted, mean ± SD	88.2 ± 6.3
FEV_1_, % predicted, mean ± SD	78.8 ± 9.5

SD, standard deviation; ECOG PS, Eastern Cooperative Oncology Group performance status; NSCLC, non-small cell lung cancer; SCLC, small cell lung cancer; ICI, immune checkpoint inhibitor; ICI–RT interval, interval between last ICI dose and radiotherapy initiation; CIP, checkpoint inhibitor pneumonitis; IMRT, intensity-modulated radiotherapy; VMAT, volumetric modulated arc therapy; V_20_, percentage of total lung volume receiving ≥20 Gy; V_5_, percentage of total lung volume receiving ≥5 Gy; MLD, mean lung dose; DLCO, diffusing capacity of the lungs for carbon monoxide; FVC, forced vital capacity; FEV_1_, forced expiratory volume in 1 second.

### 3.2 Primary endpoint

The primary study endpoint was the rate of grade 2 or higher RILI occurring within 6 months after radiotherapy. The overall incidence reached 23.1%, corresponding to 9 out of 39 patients (95% CI 11.1–39.3). This rate was significantly lower than the predefined historical control rate of 40% (one-sided exact binomial p=0.0205), meeting the final criteria of the Simon two-stage design (≤11 events among 39 patients).

Among the nine patients who developed ≥grade 2 RILI, six (15.4%) had grade 2 events and three (7.7%; 95% CI 1.6–20.9) had grade 3 events. No grade 4 or 5 RILI events were observed. For cases with grade 2 and above RILI, the median onset time was 9.1 weeks after radiotherapy. The corresponding time range spanned from 4.9 weeks to 16.6 weeks. The distribution of RILI grades across the cohort is shown in **Figure 2**.

**Figure 2:**
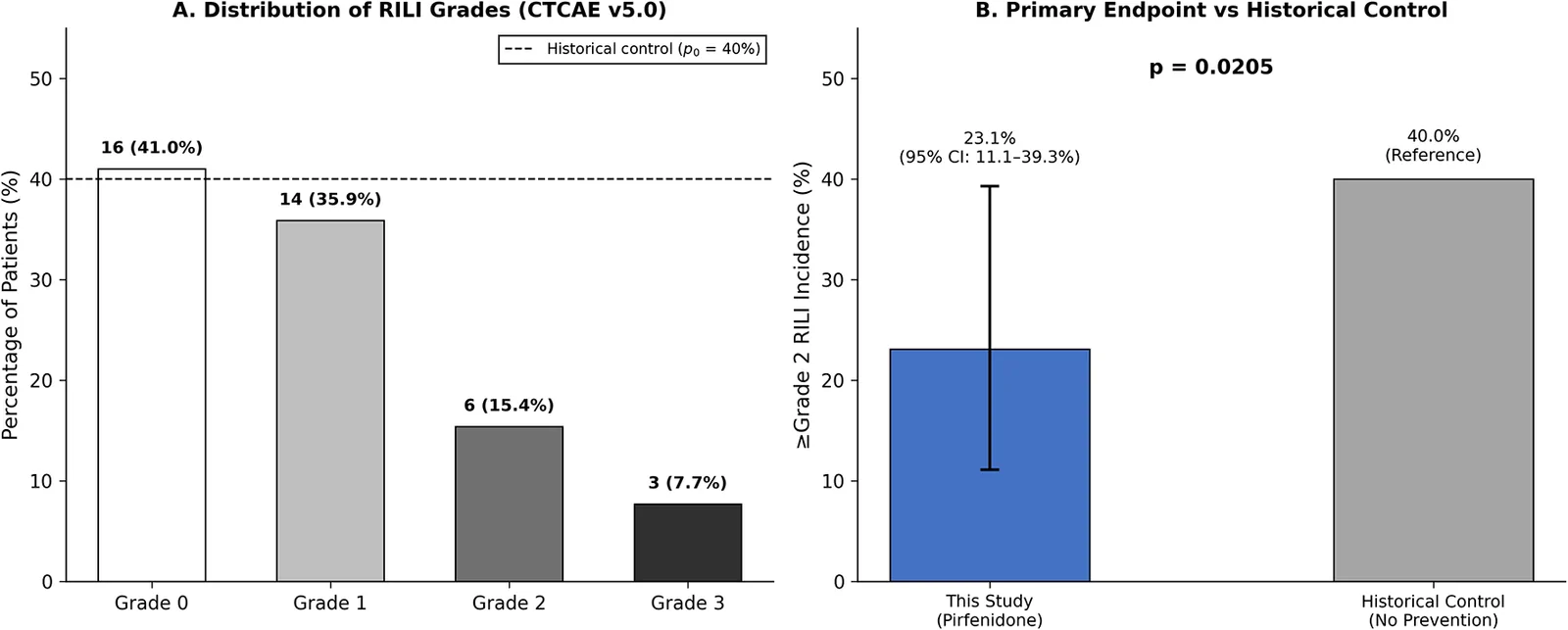
Distribution of radiation-induced lung injury (RILI) grades and primary endpoint comparison with historical control. **(A)** Maximum per-patient RILI grade within 6 months after radiotherapy in all 39 patients; no grade 4 or 5 events occurred. The dashed line marks the predefined historical control rate (p_0_ = 40%) for reference. **(B)** Incidence of ≥grade 2 RILI in the present study compared with the predefined historical control rate. The error bar denotes the exact Clopper–Pearson 95% confidence interval; the historical control is a fixed reference value and is therefore shown without a confidence interval. The p value was obtained from a one-sided exact binomial test against p_0_ = 0.40. CTCAE, Common Terminology Criteria for Adverse Events.

### 3.3 Secondary endpoints

#### 3.3.1 Pulmonary function changes

Pulmonary function was serially assessed at baseline, 3 months, and 6 months post-radiotherapy (**Table 2**; **Figures 3**, **4**). At the 3-month follow-up, DLCO% showed an average of -7.6% relative to baseline (SD 5.4). By the 6-month time point, the mean reduction in DLCO% was 6.5% (SD 5.8, p < 0.001). On the other hand, at the 3-month follow-up, the average variation in FVC% was −2.9% with a standard deviation of 3.6 (p < 0.001). At 6 months, the mean FVC% alteration reached −1.7% (SD = 3.7, p = 0.011). Individual-patient changes in DLCO% from baseline are displayed as waterfall plots at 3 months (**Figure 3A**) and at 6 months (**Figure 3B**); each bar is color-coded by RILI status, with declines exceeding 10 percentage points concentrated among patients who developed ≥grade 2 RILI.

**Table 2:** Pulmonary function changes over time.

Parameter	Baseline (N = 39)	3 Months (N = 37)	6 Months (N = 35)
All patients (N = 39)			
DLCO, % predicted (mean ± SD)	82.6 ± 13.7	74.7 ± 15.3	75.0 ± 14.8
Change from baseline	—	−7.6 ± 5.4 (*p* < 0.001)	−6.5 ± 5.8 (*p* < 0.001)
FVC, % predicted (mean ± SD)	88.2 ± 6.3	85.1 ± 7.4	85.8 ± 6.1
Change from baseline	—	−2.9 ± 3.6 (*p* < 0.001)	−1.7 ± 3.7 (*p* = 0.011)
≥Grade 2 RILI (n = 9)			
DLCO change from baseline	—	−14.6 ± 4.7	−14.3 ± 4.9
FVC change from baseline	—	−6.5 ± 4.2	−3.5 ± 4.4
<Grade 2 RILI (n = 30)			
DLCO change from baseline	—	−5.4 ± 3.4	−3.8 ± 3.0
FVC change from baseline	—	−1.8 ± 2.6	−1.1 ± 3.3

DLCO, diffusing capacity of the lungs for carbon monoxide; FVC, forced vital capacity; RILI, radiation-induced lung injury; SD, standard deviation. *p*-values were derived from paired *t*-tests comparing change from baseline. Sample sizes at 3 and 6 months reflect available follow-up data at each time point. RILI graded per Common Terminology Criteria for Adverse Events (CTCAE) v5.0.

**Figure 3:**
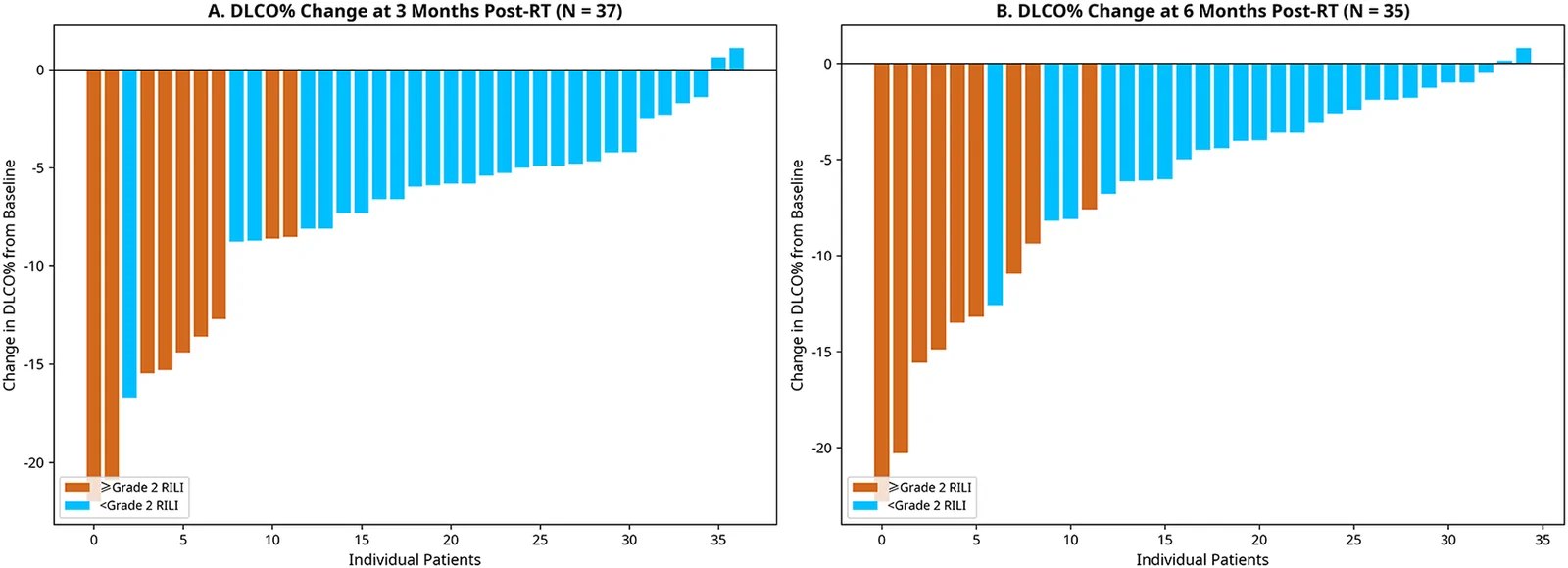
Waterfall plots of individual patient changes in DLCO% from baseline at 3 months **(A)** and 6 months **(B)** post-radiotherapy. Each bar represents one patient and shows the absolute change from that patient’s own baseline, ordered from the largest decline (left) to the smallest change or improvement (right). Orange bars denote patients who developed ≥grade 2 RILI and blue bars those who did not. Only patients with paired measurements at the respective time point are included. DLCO, diffusing capacity of the lung for carbon monoxide; RILI, radiation-induced lung injury; RT, radiotherapy.

**Figure 4:**
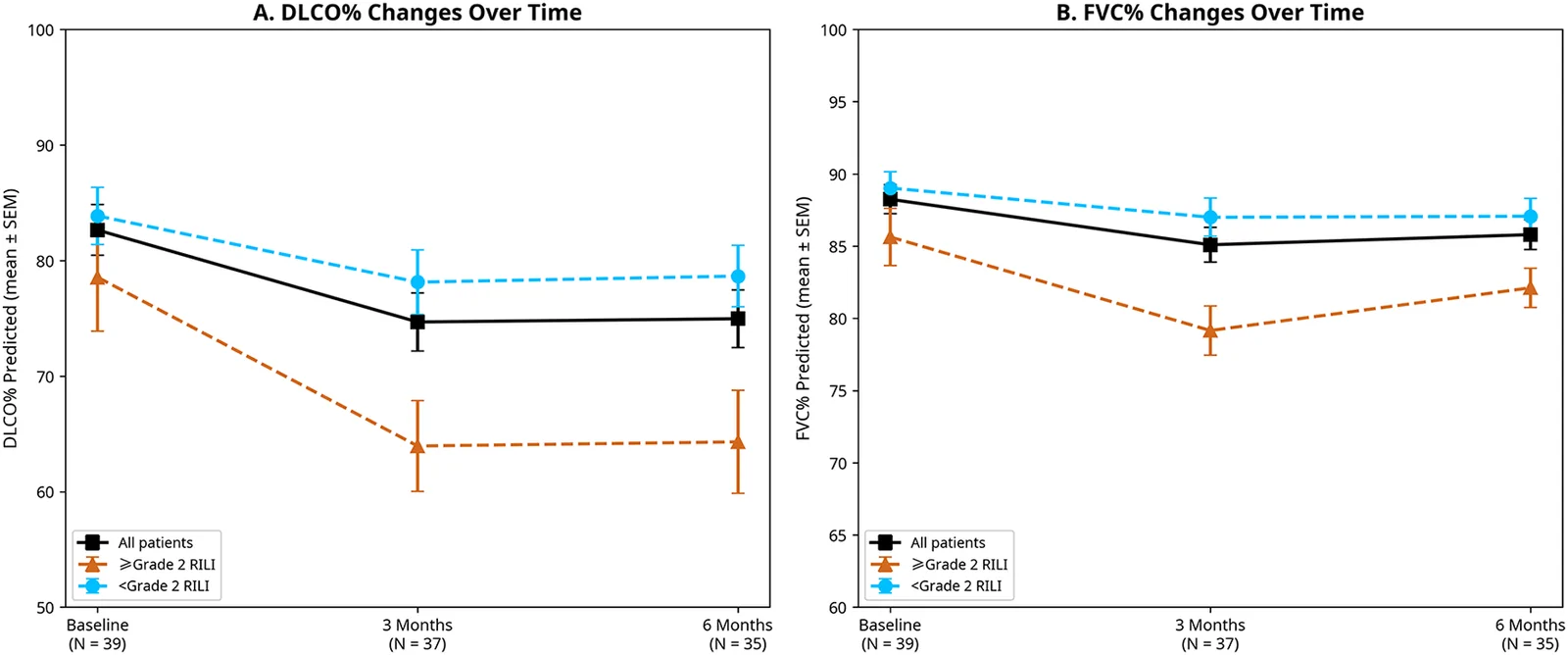
Trends in pulmonary function parameters (DLCO% and FVC%) over time, stratified by RILI severity. Mean values of DLCO% **(A)** and FVC% **(B)** at baseline and at 3 and 6 months after radiotherapy are shown for all patients (black squares, solid line), for patients who developed ≥grade 2 RILI (orange triangles, dashed line) and for those who did not (blue circles, dashed line). Error bars denote the standard error of the mean. Pulmonary function data were available for 39 patients at baseline, 37 at 3 months and 35 at 6 months. Patients were grouped according to whether they developed ≥grade 2 RILI within 6 months after radiotherapy (n = 9) or did not (n = 30). DLCO, diffusing capacity of the lung for carbon monoxide; FVC, forced vital capacity; RILI, radiation-induced lung injury.

When stratified by RILI status, patients who developed ≥grade 2 RILI exhibited a significantly greater decline in DLCO% at 3 months compared to those without ≥grade 2 RILI (-14.6% [SD 4.7] vs. -5.4% [SD 3.4]; p=0.0002).

#### 3.3.2 Exploratory subgroup analysis

Prespecified subgroup analyses are presented in **Figure 5**, together with *post hoc* analyses by age and sex. Numerically higher ≥grade 2 RILI rates were observed in patients with V20 ≥ 20%, concurrent chemotherapy use, and male sex, although none of these comparisons reached statistical significance. The incidence of ≥grade 2 RILI was 25.0% (3/12) among patients with an ICI-to-radiotherapy interval of ≤60 days and 22.2% (6/27) among those with an interval of >60 days (absolute difference 2.8 percentage points; Fisher’s exact p = 1.000). Among the 5 patients with prior grade 1 CIP, 3 (60.0%) developed ≥grade 2 RILI compared with 6/34 (17.6%) without prior CIP (Fisher’s exact p = 0.070). In the prespecified descriptive analysis by disease stage, ≥grade 2 RILI occurred in 3/11 (27.3%) patients with stage IIIA disease, 2/11 (18.2%) with stage IIIB, 3/10 (30.0%) with stage IIIC and 1/7 (14.3%) with stage IV disease. Because only nine events occurred in total, each stratum contained three or fewer events, and no formal comparisons between individual stages were undertaken; in an exploratory pooled comparison, the incidence in stage III disease (8/32; 25.0%) did not differ significantly from that in stage IV disease (1/7; 14.3%; Fisher’s exact p = 1.000).

**Figure 5:**
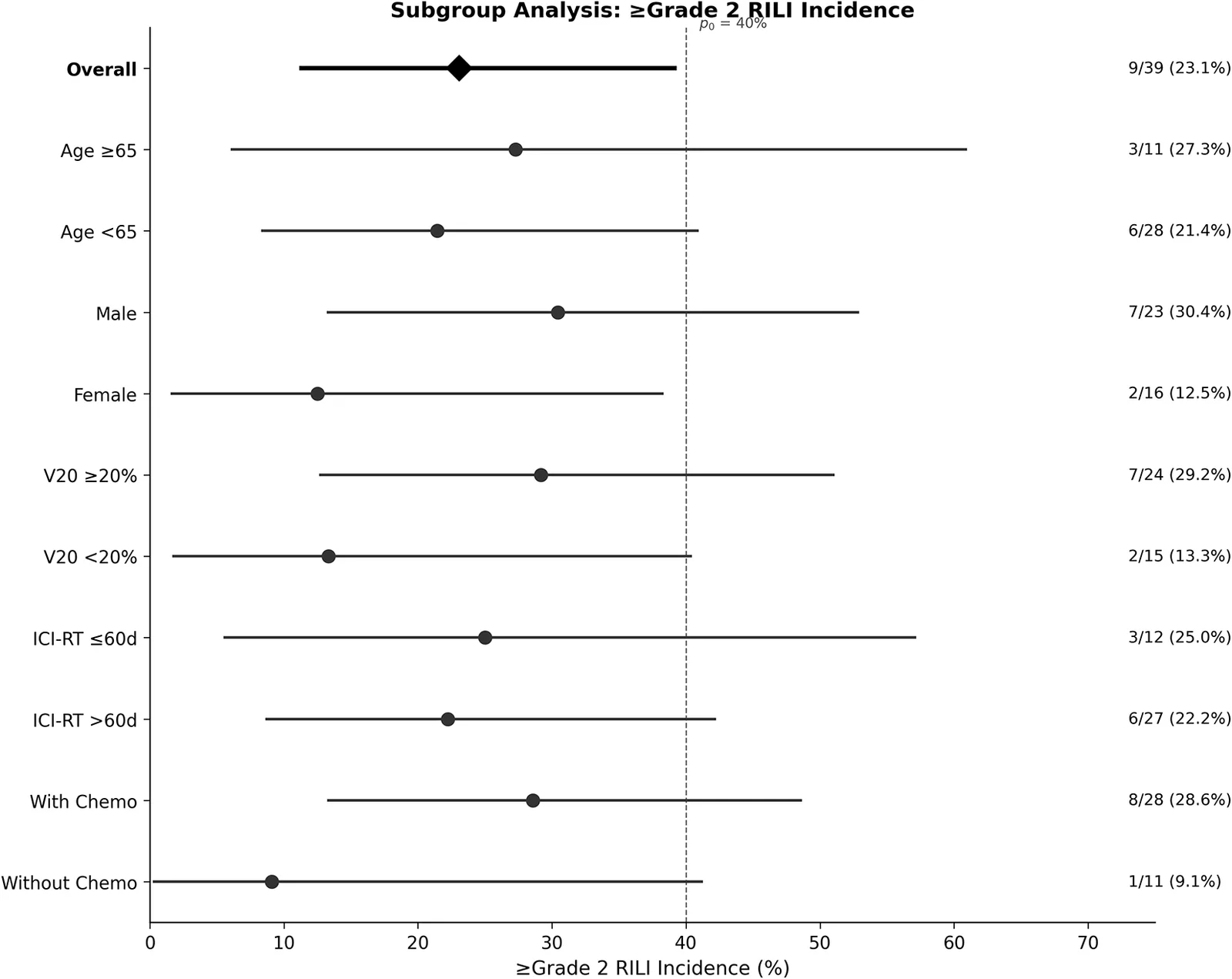
Forest plot of subgroup analyses for the incidence of ≥grade 2 RILI. Analyses by V20, ICI-to-radiotherapy interval and concurrent chemotherapy were prespecified; those by age and sex were performed *post hoc*. Markers denote the observed incidence and horizontal lines the 95% confidence intervals; the number of patients with an event, the subgroup size and the incidence are given on the right. The top row shows the overall cohort and the vertical dashed line marks the predefined historical control rate (p_0_ = 40%). All subgroup analyses were exploratory, were based on only nine events in total and were not adjusted for multiplicity. ICI-RT, interval from the last ICI dose to the start of radiotherapy; RILI, radiation-induced lung injury.

### 3.4 Safety and treatment compliance

The safety profile of prophylactic pirfenidone is summarized in **Table 3** and **Figure 6**. Pirfenidone was completed as planned by 84.6% (33/39) of patients, with a median relative dose intensity of 89.3%. Six patients (15.4%) discontinued pirfenidone prematurely, most commonly due to adverse events (n=3; **Table 3**). The radiotherapy completion rate was 89.7% (35/39). No patients required radiotherapy interruption or concurrent chemotherapy modification attributable to pirfenidone.

**Table 3:** Pirfenidone-related adverse events (N = 39).

Adverse event	Grade 1, n (%)	Grade 2, n (%)	Grade ≥3, n (%)	Any grade, n (%)
Fatigue	7 (17.9)	8 (20.5)	2 (5.1)	17 (43.6)
Nausea	11 (28.2)	5 (12.8)	0 (0.0)	16 (41.0)
Appetite loss	7 (17.9)	5 (12.8)	1 (2.6)	13 (33.3)
Diarrhea	6 (15.4)	2 (5.1)	2 (5.1)	10 (25.6)
Dyspepsia	7 (17.9)	3 (7.7)	0 (0.0)	10 (25.6)
Photosensitivity	8 (20.5)	2 (5.1)	0 (0.0)	10 (25.6)
Rash	4 (10.3)	4 (10.3)	0 (0.0)	8 (20.5)
Liver function abnormality	4 (10.3)	1 (2.6)	0 (0.0)	5 (12.8)

Adverse events were graded according to CTCAE v5.0. Patients may have experienced more than one adverse event; therefore, column totals may exceed the total number of patients.

**Figure 6:**
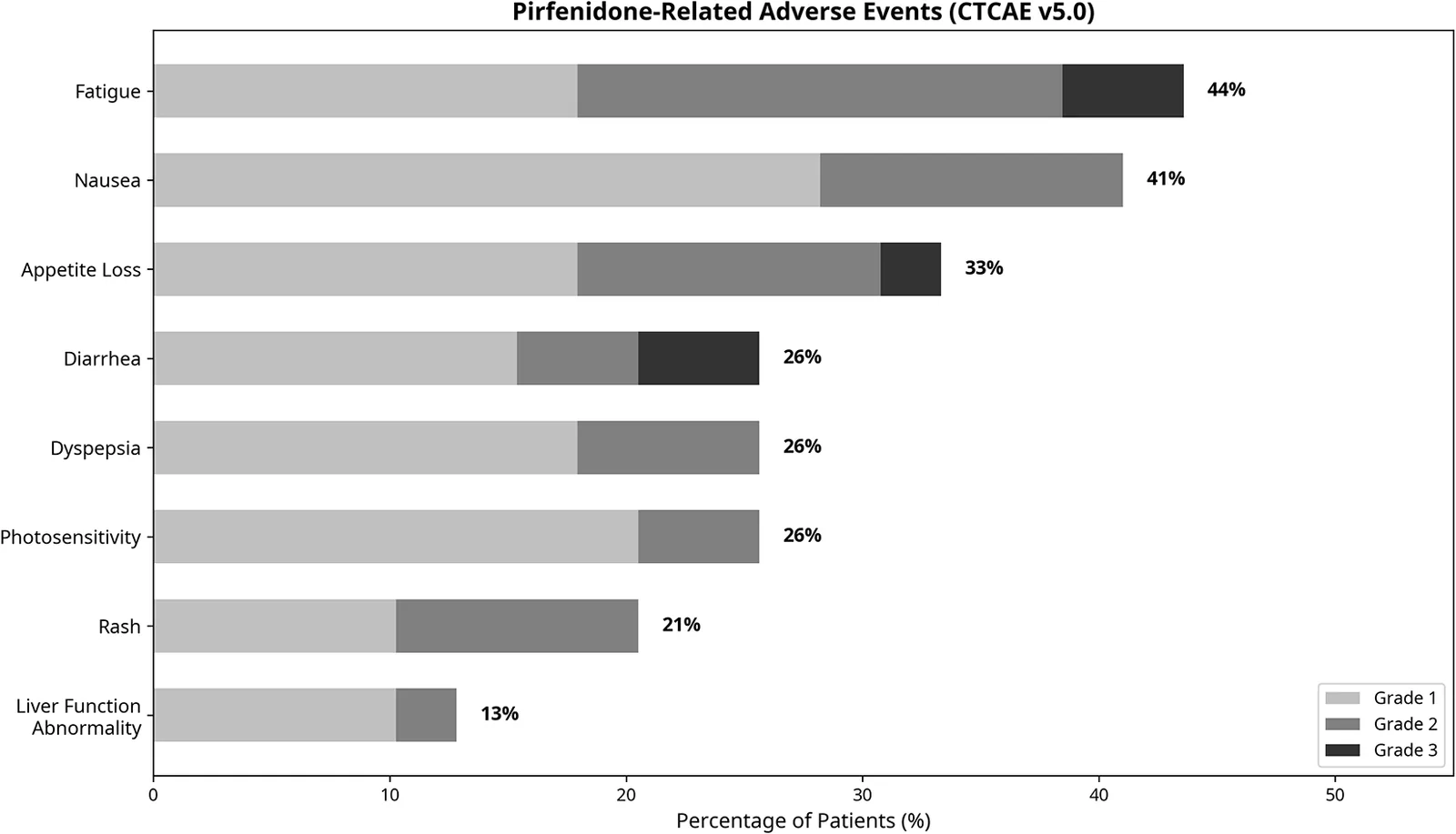
Incidence and severity of pirfenidone-related adverse events (CTCAE v5.0). Bars show the percentage of the 39 patients experiencing each event, with segments corresponding to grade 1 (light gray), grade 2 (medium gray) and grade 3 (dark gray); each patient is counted once per event type, at the highest grade recorded. No grade 4 or 5 events occurred.

## 4 Discussion

This prospective Phase II clinical study shows that preventive use of pirfenidone can lower the risk of radiation-induced lung injury in lung cancer patients who have received immune checkpoint inhibitor therapy before. The incidence of grade 2 or higher RILI in our cohort was 23.1% (95% CI: 11.1%–39.3%), reflecting a clinically meaningful and statistically significant reduction when compared with the preset historical control rate of 40% (p = 0.0205). The preventive regimen was well tolerated and there was good patient compliance; no interference with concurrent antitumor therapy was seen. As, to our knowledge, the first prospective study to evaluate antifibrotic prophylaxis in this high-risk ICI-pretreated population, our results address an important clinical gap and provide preliminary, hypothesis-generating evidence to inform future RILI-prevention strategies in thoracic oncology.

Driven by the pathological features of RILI, as well as the unique immune microenvironment in patients with prior ICI exposure, the shift from targeted treatment to preventive strategy is biologically justified. Radiation therapy triggers lung inflammation by prompting excessive reactive oxygen species production and activating the TGF-β signaling pathway. These biological processes gradually lead to irreversible fibrotic damage in the lung parenchyma (11–13, 29). Prior ICI treatment can activate the human immune system, resulting in the expansion of effector T cells and raising baseline levels of proinflammatory mediators (23, 24). This persistent immune overactivation makes patients more susceptible to radiation-induced lung injury and explains why the rate of grade ≥2 RILI remains as high as 40%–46% in this high-risk subgroup (6–8). Early administration of pirfenidone before radiotherapy helps build a protective antifibrotic microenvironment, which blunts the initial pathological cascade and breaks the self-reinforcing cycle of inflammation and fibrosis.

Our findings extend the existing evidence base and support the hypothesis that a preventive approach may be advantageous over treatment of established injury. Previous landmark studies, such as the randomized trial by Hou et al., established the efficacy of pirfenidone in mitigating established RILI (18). Retrospective analyses further prove the clinical value of this drug for patients with non-small cell lung cancer (NSCLC) (30). The inflammatory cascade will be fully activated once it starts. It is still very hard to reverse fibrotic damage in clinical work. However, our preventive regimen appears to deliver tangible functional benefits. Within six months after radiotherapy, the DLCO% of patients in our study group only dropped by 6.5%. This value is much lower than the common decline of 10% to 22% in patients with symptomatic pneumonitis (25, 26). Most previous prevention studies only enrolled patients undergoing routine radiotherapy (19, 20). Many randomized trials mainly test the effect of nintedanib and other anti-fibrotic drugs on existing radiation pneumonitis (31). This study uses a prevention method that differs from those studies. We focus on high-risk patients who have taken ICI treatment and have a higher risk of lung injury.

Risk stratification based on prior immune-related adverse events holds great clinical value, which stands out as one of the major clinical implications of this study. The results show that even with prophylactic administration of pirfenidone treatment, patients with a history of grade 1 CIP still had a high rate of grade 2 or higher RILI at 60.0%. The rate was only 17.6% in patients without a CIP history. This comparison is limited by the small sample size, with Fisher’s exact test p=0.070. The findings show that a past CIP history may suggest a clear state of immune priming in these patients, who often respond poorly to common preventive treatment. Overall, the results of this study have important clinical guiding value. For patients with a CIP history who plan to receive thoracic radiotherapy, clinicians need to arrange closer follow-up and stronger safety monitoring. At the same time, even when anti-fibrotic preventive drugs are used together, individualized preventive strategies should still be adopted. Pirfenidone has been proved to have good long-term tolerance in patients with idiopathic pulmonary fibrosis (14, 27, 28), which can also keep a good safety level when used for prevention in complex clinical situations. Gastrointestinal discomfort and mild photosensitivity are its most common side effects. These discomforts can be well controlled by supportive treatment or dose adjustment. More importantly, pirfenidone does not affect the normal delivery of thoracic radiotherapy and concurrent chemotherapy. The radiotherapy completion rate in this study was as high as 89.7%. No treatment stopped because of the study drug during the whole trial. This result further supports the above conclusion. The daily dose of 1800 mg demonstrated clinical feasibility. The overall treatment completion rate was 84.6%, which shows that preventive use of pirfenidone can be seamlessly integrated into routine outpatient treatment. Pharmacoeconomic studies also support its clinical use. Related analyses show that this drug has well-characterized tolerability in the treatment of fibrotic lung diseases (32).

Several limitations inform the interpretation of our results. First, this study adopted a single-arm, open-label design. Such a design is widely applied in phase II oncology trials, which requires rigorous outcome reporting and statistical adjustment (33). Meanwhile, adopting historical control data inevitably brings potential selection bias and evaluation bias to the research. We strictly matched baseline characteristics including lung V20 and the interval from ICI therapy to radiotherapy with historical cohorts (6, 7), to ensure group comparability. Nevertheless, unmeasured confounding factors cannot be fully ruled out, which include the continuous progress of high-precision conformal radiotherapy technology in clinical practice. Relatedly, the permitted ICI-to-radiotherapy interval of up to 6 months introduced heterogeneity in prior ICI exposure and immune-priming status, which may modulate baseline RILI risk. Although the interval was examined as a prespecified exploratory subgroup (≤60 days: 3/12 [25.0%] vs. >60 days: 6/27 [22.2%]; Fisher’s exact p = 1.000), this comparison rested on only nine events in total and was therefore underpowered to exclude a clinically meaningful difference; residual confounding by interval-related factors—such as the number of prior ICI cycles, disease stage, and radiotherapy intent—cannot be excluded and should be addressed in adequately powered randomized trials with prespecified stratification by ICI-to-radiotherapy interval. Second, although the sample size was sufficient to assess the primary endpoint, it limited the performance of rigorous multivariable analysis and prevented definitive identification of factors contributing to prophylactic failure. Third, enrolled patients presented with relatively preserved baseline pulmonary function (e.g., FEV1 > 1.0 L), which may reduce the external validity of our results and restrict extrapolation to subjects with severe underlying respiratory dysfunction. Finally, this study has no longitudinal biomarker data, such as the dynamic circulating levels of TGF-β and IL-6. So this work cannot fully explain the mechanistic relationship among pirfenidone, thoracic radiotherapy and the immune-activated tumor microenvironment.

## 5 Conclusions

In conclusion, this prospective phase II study suggests that preventive use of pirfenidone is safe and well tolerated with good clinical feasibility. For lung cancer patients who have received ICI treatment and will receive thoracic radiotherapy later, this method can lower the risk of clinically significant radiation-induced lung injury. Pirfenidone can control the abnormal inflammatory and fibrotic reaction in this high-risk group. It may confer a pulmonary protective effect without compromising standard anti-tumor treatment. This study provides proof-of-concept evidence, which explains the main underlying mechanism and provides quantitative data of treatment effect. As a single-arm phase II study, however, these findings are hypothesis-generating and require confirmation in a randomized controlled trial. The findings lay a solid foundation for large multicenter randomized phase III clinical trials in the future.

## Data Availability

The original contributions presented in the study are included in the article/**Supplementary Material**. Further inquiries can be directed to the corresponding author.
