# Supplementary material for: Prophylactic pirfenidone to prevent radiation- induced lung injury in ICI-pretreated lung cancer patients: a phase II study
Source: Front Oncol. 2026 Oct 15;16:1876642. doi: 10.3389/fonc.2026.1876642 (PMC13716608; doi:10.3389/fonc.2026.1876642)
Supplement: Supplementary file 1 [file Table1.docx]

**Supplementary Material**

**Supplementary Table 1.** Comparison of Baseline Characteristics and ≥Grade 2 Radiation Pneumonitis Incidence Between Historical Control Studies and the Present Study

| **Study** | **Design** | **Population** | **N** | **ICI Regimen** | **Prior ICI Cycles** | **RT Dose** | **V_20_ (%)** | **Concurrent Chemo (%)** | **Grading** | **≥Gr 2 RP (%)** | **Median FU** |
| --- | --- | --- | --- | --- | --- | --- | --- | --- | --- | --- | --- |
| Xiao et al. (2025) [6] | Prospective biomarker cohort, multicenter | Lung cancer with prior ICI | 71^a^ | PD-1/PD-L1 | Median: 4 | Median: 60 Gy | NR | NR | CTCAE 4.0 ≥Gr 2^b^ | 45.07 | 20.6 mo |
| Yegya-Raman et al. (2024) [7] | Retrospective, single-center | LA-NSCLC, ICI consolidation era | 335^c^ | Durvalumab consolidation | N/A (consolidation) | cCRT 60–66 Gy | Median: 26.5 | 100 | CTCAE ≥Gr 2 | 31.4^d^ | 39.6 mo |
| This study | Prospective, single-arm, single-center | ICI-pretreated lung cancer | 39 | PD-1/PD-L1 (prior treatment) | Median: 5 (2–10) | Median: 60 Gy (MLD: 8.8 Gy) | Mean: 21.0 (SD 4.3) | 71.8 | CTCAE v5.0 ≥Gr 2 | 23.1 | 6 mo |

^a^ ICI-pretreated subgroup of 214 total patients enrolled in the study. The overall cohort symptomatic RP rate was 35.05% (75/214).

^b^ Graded according to CTCAE 4.0. Defined as “symptomatic radiation pneumonitis” (≥grade 2) by the original authors.

^c^ ICI-era cohort (2017–2021) within a total study population of 783 patients. The pre-ICI era cohort (2011–2017; n = 448) had a 1-year ≥grade 2 pneumonitis rate of 20.1%. Median V_20_ in the ICI-era cohort was 26.5% and median MLD was 15.3 Gy.

^d^ Reported as 1-year cumulative incidence (IPTW-adjusted subdistribution hazard ratio vs pre-ICI era: 2.03; 95% CI: 1.53–2.70; *p* < 0.001). Cut-point analysis within the ICI-era cohort identified a V_20_ threshold of 28%, above which the rate reached 46.0% versus 19.8% below (*p* < 0.001).

**Abbreviations:** cCRT, concurrent chemoradiotherapy; CTCAE, Common Terminology Criteria for Adverse Events; FU, follow-up; Gr, grade; ICI, immune checkpoint inhibitor; LA-NSCLC, locally advanced non-small cell lung cancer; MLD, mean lung dose; mo, months; N/A, not applicable; NR, not reported; RP, radiation pneumonitis; RT, radiotherapy; SD, standard deviation; V_20_, percentage of total lung volume receiving ≥20 Gy.
